# The perirhinal cortex and conceptual processing: Effects of feature-based statistics following damage to the anterior temporal lobes

**DOI:** 10.1016/j.neuropsychologia.2015.01.041

**Published:** 2015-09

**Authors:** Paul Wright, Billi Randall, Alex Clarke, Lorraine K. Tyler

**Affiliations:** Centre for Speech, Language and the Brain, Department of Psychology, University of Cambridge, Downing Street, Cambridge CB2 3EB, United Kingdom

**Keywords:** Perirhinal cortex, Anterior temporal lobe, Concepts, Semantics, Patients, Confusability

## Abstract

The anterior temporal lobe (ATL) plays a prominent role in models of semantic knowledge, although it remains unclear how the specific subregions within the ATL contribute to semantic memory. Patients with neurodegenerative diseases, like semantic dementia, have widespread damage to the ATL thus making inferences about the relationship between anatomy and cognition problematic. Here we take a detailed anatomical approach to ask which substructures within the ATL contribute to conceptual processing, with the prediction that the perirhinal cortex (PRc) will play a critical role for concepts that are more semantically confusable. We tested two patient groups, those with and without damage to the PRc, across two behavioural experiments – picture naming and word–picture matching. For both tasks, we manipulated the degree of semantic confusability of the concepts. By contrasting the performance of the two groups, along with healthy controls, we show that damage to the PRc results in worse performance in processing concepts with higher semantic confusability across both experiments. Further by correlating the degree of damage across anatomically defined regions of interest with performance, we find that PRc damage is related to performance for concepts with increased semantic confusability. Our results show that the PRc supports a necessary and crucial neurocognitve function that enables fine-grained conceptual processes to take place through the resolution of semantic confusability.

## Highlights

• We relate damage to subregions of the ATL to conceptual processing.
• Damage to the PRc, within the ATL, correlates with behavioural performance.
• Damage to PRc results in worse performance for semantically confusable concepts.
• PRc enables processing of fine-grained semantic knowledge.

## 1 Introduction

It is widely acknowledged that conceptual knowledge – our knowledge of people, places and entities – is subserved by a distributed neural system which includes the anterior temporal lobes (ATL). These regions feature in a number of neurobiological models of semantic knowledge, and are central to the hub and spoke model (Patterson et al., 2007; Rogers and Patterson, 2007) which is based primarily on data from patients suffering from the neurodegenerative disease Semantic Dementia (SD). SD is characterised by a progressive deterioration of conceptual knowledge in the context of well-preserved cognition. Patients with SD typically have semantic deficits in all modalities, and for all kinds of concepts, leading to the claim that the ATL is an amodal semantic hub in which different types of information relevant to semantic representations – e.g. sensory, motor and linguistic – converge (Patterson et al., 2007).

However, since the pathology in SD involves widespread damage to the ATL (amoungst other regions, Brambati et al., 2009; Mion et al., 2010; Noppeney et al., 2007) as the disease progresses (Bright et al., 2008), it has not been entirely clear which specific regions within the ATL contribute to the patients' semantic memory deficits. Acknowledging this lack of clarity, Binney et al. (2010) carried out a study in which they differentiated between a series of regions in the ATL. They defined ROIs which covered the lateral to medial extent of the ATL – including the temporal pole, superior temporal gyrus, middle temporal gyrus, inferior temporal gyrus, fusiform gyrus and the parahippocampal gyrus - and reported that the anterior ventral and inferolateral temporal lobe regions were essential for performance on semantic tasks.

In contrast to reports of a ventral and lateral anterior temporal focus for semantic effects in the ATL, the anteromedial regions of the ATL are also claimed to be critically involved in semantic computation, as revealed in the reports of category-specific semantic deficits in patients with anteromedial temporal lobe damage (Warrington and Shallice, 1984) and in a variety of subsequent behavioural and neuroimaging studies with healthy participants (Barense et al., 2010; Clarke and Tyler, 2014; Kivisaari et al., 2012; Moss et al., 2005; Taylor et al., 2006, 2009; Tyler et al., 2013, 2004; Wang et al., 2010). Patients who have category-specific semantic deficits know the category of an object, but they are exceptionally poor at differentiating between similar objects within a category. Moreover, this pattern is most pronounced for living things, especially animals (Moss et al., 2005, 1998, 1997; Warrington and Shallice, 1984).

Moss et al. (2005) linked these findings to a hierarchical neurobiological system of increasing feature complexity along the ventral stream (Ungerleider and Mishkin, 1982) in which simple visual features are processed in more posterior sites, with increasingly complex conjunctions of features more anteriorly, culminating in the apex of the stream – the perirhinal cortex (PRc) – which performs the most complex feature conjunctions (Barense et al., 2012; Cowell et al., 2010; Murray and Bussey, 1999; Murray et al., 2007). Moss et al. (2005) argued that these neural properties of the PRc provided the basis for the fine-grained analysis required for differentiating between highly similar concepts. Related research has found that when the PRc is damaged, patients have a category-specific deficit for living things, whereas patients with antero-lateral temporal lobe damage have a generalised semantic impairment and no category-specific impairment (Moss et al., 2005; Noppeney et al., 2007; Rogers and Patterson, 2007) (also see Bruffaerts et al., 2014 for a case with a living things deficit and spared PRc). The relationship between antero-medial temporal lobe structures and semantic processing has been further supported by neuroimaging studies with healthy volunteers that show living things preferentially engage the antero-medial temporal lobes (Moss et al., 2005; Taylor et al., 2006).

However, Tyler and colleagues argue that the association between living things deficits and increased activity for living things in the PRc is not due to category membership *per se* (Moss et al., 2005; Taylor et al., 2006; Tyler et al., 2013, 2004). Indeed, category effects in neuroimaging are not only observed in the PRc but also in more posterior regions (see Martin, 2007). Instead, they propose that effects for living things in the PRc are due to the extent to which members within a category are confusable. They assume a componential model of conceptual representations in which concepts are made up of smaller elements of meaning, referred to as features, properties or attributes (Cree and McRae, 2003; Gotts and Plaut, 2004; McRae et al., 1997; Mirman and Magnuson, 2008; Randall et al., 2004). In this type of model, features that are shared by many objects provide the basis for categorization (Smith and Medin, 1981), while those that are distinctive enable similar objects to be differentiated from each other (Cree and McRae, 2003; Taylor et al., 2012, 2008). According to property norm data, living things have more shared features and are therefore more highly confusable than members of other categories (Devereux et al., 2014; Keil, 1986; Malt and Smith, 1984; McRae et al., 1997; Randall et al., 2004), making them more dependent upon PRc function in order to differentiate one living thing from another, a prediction that has been supported by recent data from an fMRI study (Tyler et al., 2013). In contrast, category effects in the fusiform are claimed to be due to overlap in shared features, providing the basis of category structure (Tyler et al. 2013).

Conceptual structure measures derived from one feature-based model, the Conceptual Structure Account (CSA; Taylor et al., 2011; Tyler and Moss, 2001), which captures the statistical properties within and between concepts, have been widely used to probe the details of conceptual representation in behavioural, modelling and brain imaging studies (Clarke et al., 2013; Randall et al., 2004; Taylor et al., 2012, 2008; Tyler et al., 2013). Specifically, a feature statistic reflecting differentiation between highly similar objects, thus enabling object-specific representations, was associated with bilateral PRc activity in a recent fMRI study (Tyler et al., 2013). In the current paper, we manipulate semantic confusability to ask whether damage to the PRc impairs performance in the conceptual processing of concepts that require a high degree of within-category differentiation. To do this we developed two behavioural studies which measured different aspects of conceptual representation and were appropriate for brain-damaged patients – picture naming and word-picture matching. We tested patients who had a single lesion that was confined within the ventral temporal lobe, occipital lobe or temporal pole. These patients were divided into two groups depending on whether they had damage including the PRc, or whether the PRc was intact, and performance was compared in the different tasks to assess the impact of PRc damage on conceptual processing. As damage was not restricted to the PRc, but also effected other ventral anterior temporal lobe (vATL) structures, we also relate the degree of damage in anatomically defined vATL substructures (such as the PRc, fusiform etc.) to performance in order to test specific claims about the nature of semantic processing in specific vATL substructures.

Across the experiments we tested the impact of semantic confusability in three ways. First, we tested picture naming performance for different categories, with the prediction that damage to the vATL, and the PRc specifically, will result in impaired performance for living things which have a greater degree of within-category confusability than nonliving things. Second, we tested the relationship between key conceptual structure statistics and naming performance. Three measures were derived from our property norm data (Devereux et al., 2014) to capture the internal conceptual structure of the objects, (a) mean distinctiveness, (b) correlational strength and (c) the relationship between distinctiveness and correlational strength (‘correlation×distinctiveness’; see Taylor et al., 2012 for further details). Mean distinctiveness is calculated as the average distinctiveness of all the features in a concept. When a concept has many shared features, distinctiveness will be low and when it has many distinctive features, it will be high. The correlational strength of a concept is the average of all significant pairwise correlations between the shared features (i.e. those occurring in at least three concepts) of a concept. High correlational strength indicates that the features in a concept tend to co-occur and is a measure that is crucial to the formation of categories. The ‘correlation×distinctiveness’ measure aims to capture the relationship between correlational strength and a concept's distinctive and shared features. The measure is the unstandardised slope of the regression line describing the scattergraph of each concept's features with correlational strength and distinctiveness on the axes (see Taylor et al., 2012, pp. 366–367 for a full description of this measure). Following our previous studies, we predicted that objects which have many shared features and few weakly correlated features (e.g. the typical conceptual structure of living things and measured by the ‘correlation×distinctiveness’ measure) would be most affected by damage to the ventral anterior temporal lobe and in particular to the perirhinal cortex. Further, we predict that neither mean distinctiveness nor correlational strength would influence behaviour for patients with damage to the PRc, as mean distinctiveness (or sharedness, as its inverse) is associated with the posterior fusiform gyrus (Tyler et al., 2013) which is not damaged in these patients, and correlational strength is important for the representation of categories, an area where we do not expect this group to have any difficulty. Third, we manipulated the semantic confusability of concepts in a word-picture matching paradigm with the prediction that damage to the PRc will result in impaired performance in distinguishing between semantically close words and pictures.

## 2 Methods

### 2.1 Participants

Fourteen patients were recruited via the Cambridge Cognitive Neuroscience panel (MRC Cognition and Brain Sciences Unit, Cambridge, UK). Inclusion criteria were: lesion confined within the ventral temporal lobe, occipital lobe or temporal pole (referred to as the ventral stream), one lesion only, high resolution T1-weighted MR image available, able to give informed consent and perform cognitive testing (no significant visual, auditory or motor impairments). All patients scored a minimum of 26/30 on the Mini Mental State Examination (Folstein et al., 1975) or 30/36 on Raven's Coloured Progressive Matrices (Raven, 1995).

**Fig. 1:**
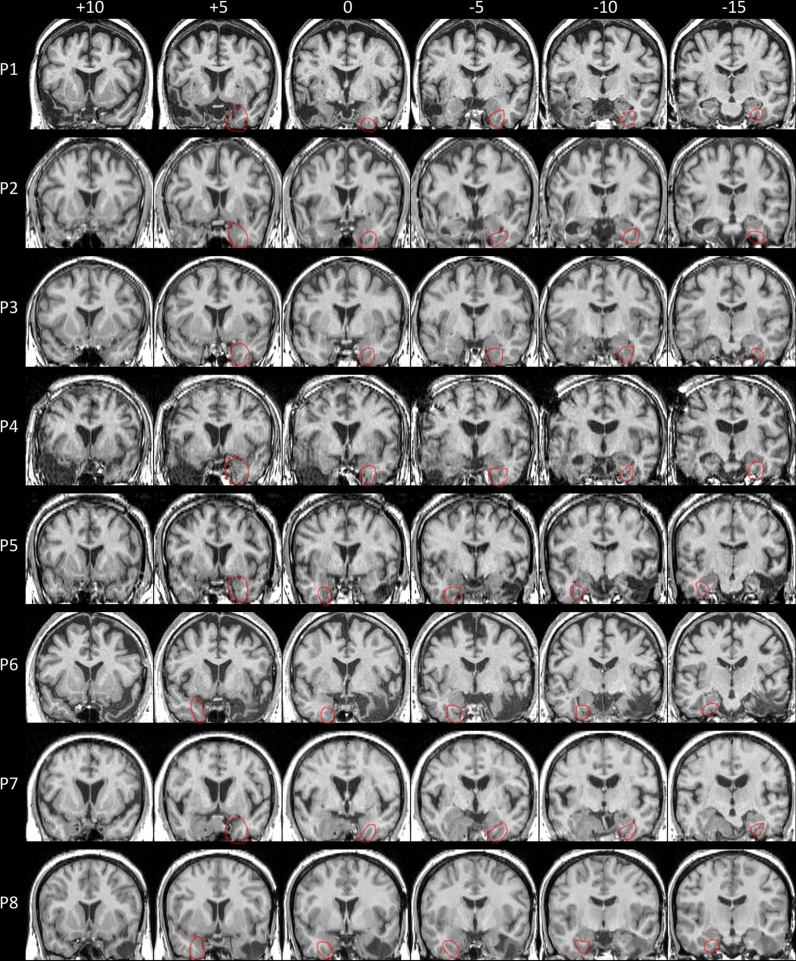
The vATL-damaged patients' lesions shown on T1-weighted structural scans normalised to MNI space. The perirhinal cortex in the intact, contralesional hemisphere is indicated in red. See Table 1 for lesion descriptions. MNI *y* and *z* coordinates are reported above each section. Images are shown in neurological convention with patient's left on image left.

**Fig. 2:**
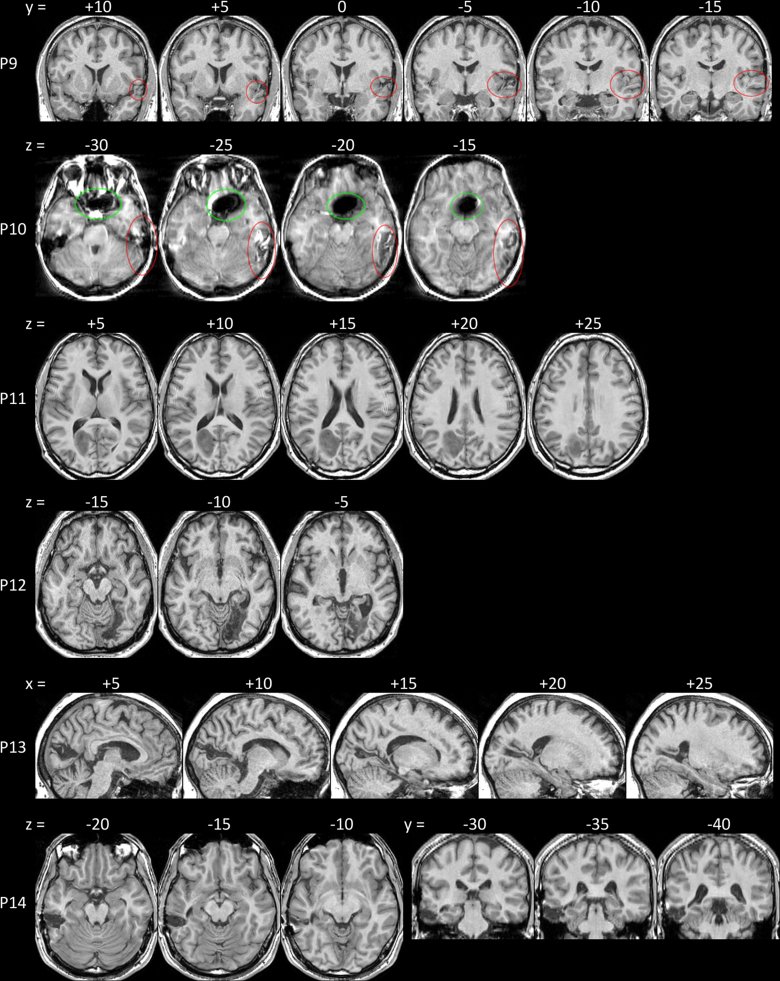
The vATL-intact patients' lesions shown on T1-weighted structural scans normalised to MNI space. For P9 & P10, the lesions are circled in red to aid location. Green circle: image artefact caused by aneurism clip. See Table 1 for lesion descriptions. MNI *y* and *z* coordinates are reported above each section. Images are shown in neurological convention with patient's left on image left.

Table 1 describes the patients' demographic and lesion information. Patients were divided into two groups based on whether the lesion included the PRc or whether the PRc was intact. As damage to the PRc will invariably lead to damage to other vATL substructures the two groups are defined as vATL-damaged and vATL-intact respectively. As Table 1 shows, the vATL-damaged patients had damage to the PRc and varying degrees of damage to other vATL structures including the fusiform, inferior temporal, middle temporal, temporal pole and entorhinal cortex. Patients in the vATL-intact group all had lesions in the ventral stream that spared the perirhinal cortex, and all were posterior to the perirhinal cortex except P9, whose lesion was in the dorsal temporal pole. Figs. 1 and 2 show the location of each patient's lesion.

In addition to the 14 patients, we recruited mature, healthy control participants to obtain baseline scores for each experiment. Control participants were aged between 50 and 75 years, had no history of neurological or psychiatric illness and scored a minimum of 26/30 on the Mini Mental State Examination or 30/36 on Raven's Coloured Progressive Matrices. The two experiments were run at separate times and four patients were unavailable for the second round of testing. Each experiment had its own set of controls. There were 15 controls (8 female) in Experiment 1 with a mean age of 58 years (SD=4.6 years), and 14 controls (10 females) in Experiment 2 with a mean age of 67 years (SD=5.1 years).

Both experiments were given ethical approval by Cambridge Central Research Ethics Committee (for patients) and University of Cambridge Psychology Research Ethics Committee (for healthy controls). Each participant gave written, informed consent before participating.

### 2.2 Materials and procedures

#### 2.2.1 Experiment 1: Picture naming

In this experiment participants named a set of common objects at the basic level (e.g. *hammer, apple*) a task that requires unique identification of an object. In similar studies with healthy people (Taylor et al., 2012) we related different conceptual structure variables to naming performance to determine which variables affected naming accuracy. Here we ask whether conceptual structure measures which measure the confusability of an object within its category will have differential effects on naming accuracy as a function of whether a patient has intact or damaged ventral anterior temporal lobe, in particular the perirhinal cortex.

##### 2.2.1.1 Procedure and stimuli

Participants named 207 common, familiar objects as quickly as possible at the basic level. Each trial began with a fixation cross on the screen for 1000 ms followed by a picture presented for 2000 ms. Participants were asked to name the picture as quickly and accurately as possible and, if they did not know an item, to make a guess. Responses were noted and recorded for further reference. There was no time out, as a new trial was only presented after a response had been made.

The 207 items were taken from the 302 items reported in Taylor et al. (2012). The items were colour photographs of familiar concrete concepts presented in isolation on a white background. Each object was associated with feature norm data obtained from an extensive feature norming study (Devereux et al., 2014), from which we calculated three conceptual structure measures: (a) mean distinctiveness, (b) correlational strength and (c) the relationship between distinctiveness and correlational strength (‘correlation× distinctiveness’).

Each stimulus could be placed easily into a superordinate category (e.g. animal, tool etc.), and was chosen to elicit a single-word response. All pictures could be reliably identified, as shown by pretests with an independent group of 20 participants, where they were asked to name each picture. Naming and concept agreement for all items included in the current study exceeded 70% (i.e. more than 70% of participants responded with the correct name or concept respectively). Within the stimuli were sets of animals (33 items), fruit and vegetables (33 items), tools (25 items) and vehicles (18 items) that were matched on naming agreement (*F*(3,105)=2.38; *p*>0.05) and concept agreement (*F*<1). In a further pretest, 15 healthy volunteers were asked to rate the pictures for their exemplarity. The participants were shown the picture with its label and asked to rate on a scale of 1–7 how good an example the picture was of the concept label. Fruit and vegetables had the highest exemplarity (*F*(3,105)=16; *p*<0.05) and animals had the lowest familiarity (*F*(3,105)=6.65; *p*<0.05 (Table S1).

The objects were presented in two blocks, with each block matched for the number of animals, fruit and vegetables, tools and vehicles. Blocks were also matched on the frequency and length of the word to be named, visual complexity, exemplarity and familiarity of the pictures. The items were pseudo-randomised such that there were no more than two consecutive items from the same superordinate category.

##### 2.2.1.2 Statistical analyses

We performed two distinct analyses on the picture naming data, testing (a) for effects associated with different categories of object and (b) for the influence of conceptual structure statistics on picture naming. For both of these analyses we focussed on naming accuracy. Accurate responses were those where the given name matched that from the property norms or a synonym.

Data were analysed using IBM SPSS Statistics v.21 (IBM UK Ltd., Portsmouth, UK). Interactions between group and each experimental manipulation were tested using repeated measures ANOVA with group as a between-subjects factor and experimental manipulations as within-subjects factors. Unless stated otherwise, follow-up t tests and correlations were run using one-tailed significance, since we had *a priori* predictions that damage to the PRc would result in lower basic naming accuracy scores, and that ROI correlations between damage and accuracy would be negative.

#### 2.2.2 Experiment 2: Word-picture matching

In this experiment we used a picture–word matching paradigm and manipulated the conceptual similarity between word and picture to test the hypothesis that word-picture pairs that are highly confusable will be disproportionately difficult for patients who have damage to the ventral anterior lobe, in particular the perirhinal cortex.

##### 2.2.2.1 Procedure and Stimuli

Participants were presented with a written word that was followed by a picture and asked to judge if the word and picture matched. Participants were seated a comfortable distance from a computer screen with a button box in front of them. They were instructed to press the yes button when the word and picture matched (cat (word)/CAT (picture); spade (word)/SPADE (picture)) and the no button when they did not match (cat/DOG; spade/RAKE) as quickly and as accurately as possible. Each trial began with a blank screen for 750 ms. The word then appeared in black lower case letters on a white background for 750 ms. There was a further blank screen for 200 ms and the picture appeared for 1000 ms. Pictures were all single concrete concepts presented in isolation on a white background. There was a time out of 2500 ms. There were 120 trials where the word and picture did not match, and a further 120 filer trials where the word and picture matched. These filler items were used to control for the number of yes and no responses, and were not included in the analysis which only considers the non-matching trials.

In order to test our predictions we manipulated the degree of conceptual similarity between the word and picture according to calculations from the CSLB property norms (Devereux et al., 2014). Reading a word (e.g. ‘crab’) activates the shared and distinctive features associated with this word (Cree et al., 2006; Randall et al., 2004). Viewing an object which has a similar meaning (e.g. ‘lobster’) will activate much of the same semantic information. In order to judge whether the word and picture are the same critically requires access to fine-grained distinctive information that can differentiate them. We predict, on the basis of our previous research (Clarke and Tyler, 2014; Kivisaari et al., 2012; Moss et al., 2005; Taylor et al., 2006, 2009; Tyler et al., 2013), that access to fine-grained semantic properties involves the perirhinal cortex. The experiment tested this hypothesis by manipulating the semantic distance between the word and picture (close and distant relationship) and the domain of the word-picture pairing (living, non-living). We used a factorial design with 30 items in each of the four conditions: living close; living distant; nonliving close and nonliving distant. Semantically close items were those which shared a large number of semantic properties (e.g. *panther and tiger, trombone and trumpet).* Semantically distant items, while belonging to the same semantic category, shared fewer features (e.g. *beaver and tiger, bagpipes and trumpet).* All items represented concrete concepts and were selected from the 638 concepts in CSLB norms (Devereux et al., 2014). We calculated the semantic distance between word and picture by using the cosine between the two concept production frequency vectors (McRae et al., 2005). The mean cosine similarity was 0.69 for close items and 0.20 for distant items, with no difference between living and nonliving things (see Table S2). Word length and word familiarity were matched across domain and by semantic distance (length: domain, *F*(1,116)=1.89, *p*>0.1, semantic distance, *F*<1; familiarity: domain, *F*(1,116)=2.57, *p*>0.1; semantic distance, *F*<1), with no interactions (*F*s<1). Picture exemplarity and visual familiarity were also matched across domains and semantic distance (exemplarity: domain, *F*<1; semantic distance, *F*(1,116)=3.00, *p*>0.05; familiarity: domain, *F*<1; semantic distance, *F*(1,116)=2.12, *p*>0.1) with no interactions (*F*s<1). Word familiarity measures were taken from the MRC psycholinguistic database (Wilson, 1988) or pretests conducted with an independent set of healthy controls. Other measures were derived from pretests conducted with healthy controls. Items were pseudorandomised such that there were no more than five consecutive same or different judgements, living or nonliving items, close or distant items.

##### 2.2.2.2 Statistical analyses

Analyses were performed as described for Picture naming.

### 2.3 Imaging

For each patient, high resolution structural MRI scans were obtained. Scans were acquired using a T1-weighted sequence, with in-plane resolution of 1 mm and slice thickness of 1–2 mm. Images were normalised to Montreal Neurological Institute (MNI) space using unified segmentation and normalisation (Ashburner and Friston, 2005) in SPM8 (Wellcome Trust Centre for Neuroimaging, London, UK).

### 2.4 Lesion definition

Patients' lesions were marked on the native space structural scan using MRIcron (Rorden, C., www.mricro.com). The borders of the lesion were defined in the plane of acquisition (usually axial) and then reviewed in the other planes and adjusted if needed. Where the border of the lesion was unclear, e.g. a graded reduction in contrast in white matter at the edge of the lesion, the border was set at the approximate half way point of the graded area. Landmarks in the intact, contralesional hemisphere were used to guide identification of damaged tissue. Where tissue changed position following damage (e.g. the grey matter overlying the amygdala shifted ventrolaterally in P6) the lesion definition took this movement into account. Where the ventricles were enlarged, they were marked as damaged where they expanded into lost tissue. In the case of P7, where the lesion was a meningioma rather than a stroke or resection, lesion definition was more difficult because the difference in contrast between healthy and damaged tissue was more subtle. However, a border between intact and damaged tissue was identified by carefully comparing the three orthogonal planes and noting the position of intact landmarks.

In the resulting lesion image, damaged voxels had a value of 1 and intact voxels 0. The native space lesion images were warped to MNI space using the normalisation parameters. Warping used trilinear interpretation and was followed by binarisation using a threshold of 0.5. This in effect applied very slight smoothing, sufficient to remove any fine variations orthogonal to the plane of lesion definition.

### 2.5 ROI definitions

A critical question in this study is whether specific anatomically defined structures within the anterior temporal lobe, such as the perirhinal cortex, make distinct contributions to semantic processing. To address this issue, we defined regions of interest (ROIs) in the anterior temporal lobe in order to obtain measures of damage in distinct regions. Using an ROI approach, Binney et al. (2010) showed that the middle and inferior temporal gyri (MTG and ITG) and fusiform gyrus all contribute to some extent to semantic processing. We adapted and extended this approach by using the latest protocols to identify and delineate the perirhinal cortex from neighbouring cortices. The perirhinal, entorhinal cortex, temporal pole and fusiform gyrus were defined using landmarks described by Kivisaari et al. (2013). The inferior and middle temporal gyri (ITG and MTG) were defined according to Tzourio-Mazoyer et al. (2002) and Rademacher et al. (1992). The fusiform, ITG and MTG ROIs were truncated posteriorly at the most posterior coronal section containing the perirhinal cortex so these ROIs covered the same anterior–posterior extent. The borders used in defining each ROI are summarised in Table 2 (for full details on how borders vary at different coronal sections and how individual anatomic variations were handled see Kivisaari et al. (2013), Insausti et al. (1998), Tzourio-Mazoyer et al. (2002) and Rademacher et al. (1992).

**Fig. 3:**
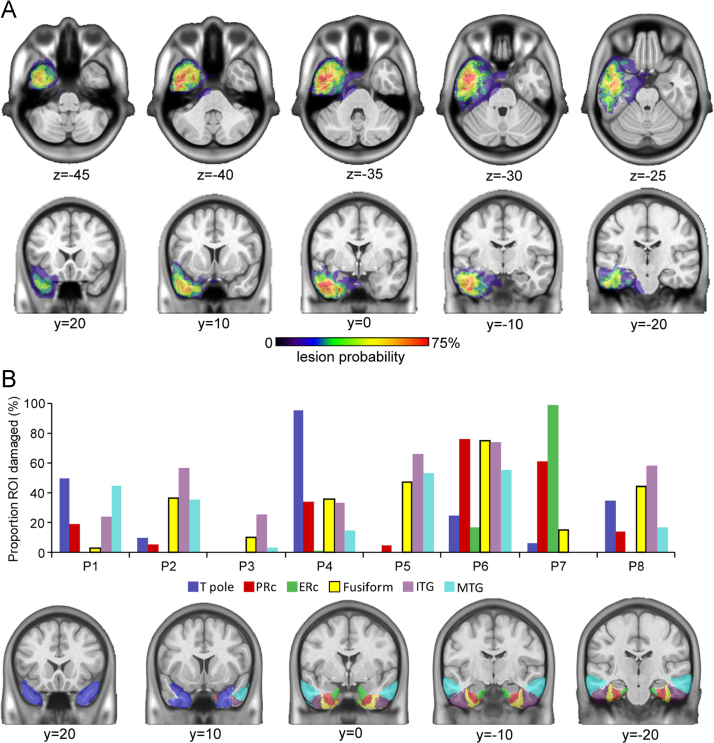
Damage in the anterior temporal lobes for the vATL-damaged group and anatomical ROIs. A: Lesion probability maps. For illustration, lesions in either hemisphere are shown on the left, overlaid on MNI atlas brain. The peak of damage occurs in anterolateral and anteromedial regions (including the PRc) in sections from +10 to −10 mm. B: Distribution of damage in vATL-damaged patients across the anatomical ROIs. Columns show the proportion of voxels damaged in each ROI, that are shown below overlaid on an MNI atlas brain.

ROIs were drawn on normalised high resolution structural scans from 15 healthy control participants from an independent study (Tyler et al., 2013). For each ROI, the 15 drawn images were combined to create a probability map. The probability maps for the six ROIs were then combined to create an atlas image, with each voxel assigned to the ROI with the highest probability (voxels where two ROIs tied for first place were left undefined). The resulting atlas ROIs (see Fig. 3b, Section 3) were then used in analysis of patients' lesions. The perirhinal cortex ROI aligned well with previously published probabilistic atlases (Devlin and Price, 2007; Holdstock et al., 2009) and the ITG and MTG aligned with the Harvard–Oxford atlas (Desikan et al., 2006). The lateral border of the fusiform gyrus ROI aligned with that of the Harvard–Oxford atlas, but the medial border, shared with the perirhinal cortex, was shifted laterally according to the definitions in Insausti et al. (1998).

The distributions of patients' lesions were quantified by extracting the mean value of each patient's lesion image within each ROI. In the lesion image, damaged voxels have a value of 1 and undamaged voxels a value of 0. The mean value over all the voxels in an ROI translates to the proportion of the ROI that is damaged. These extracted scores were used to describe the relative damage across structures in the anterior temporal lobe and in analyses that aimed to identify whether behavioural performance related to integrity of specific regions.

## 3 Results

### 3.1 Patients' lesions

The combined lesion probability map for the patient group with damage to the PRc, but also affecting the vATL (vATL-damaged group), is shown in Fig. 3A, with the distribution of lesion location in the anterior temporal lobes shown in Fig. 3B, highlighting that the group includes patients with additional medial damage (e.g. P7), lateral damage (e.g. P2 and P5) and both (e.g. P6). Note that one patient had primarily white matter damage (in particular P3), which is not quantified by the grey matter-based ROIs. Further, only two patients have damage to the ERc so this ROI was excluded from all correlational analyses. Lesion probability is not shown for the vATL-intact group, since their lesions are more heterogeneous (Fig. 2). In order to rule out differences between the two groups due to total lesion volume, lesion volumes were calculated by counting the voxels in the binary lesion masks (Table 1). There was no significant difference between the groups (vATL-damaged=14.96 cm^3^, vATL-intact=14.06 cm^3^, *t*(12)=0.16, *p*>0.5).

Because the ROIs used here border one another and are affected by lesions that cross anatomical boundaries, we tested the correlations between the % damage values for each pair of ROIs (Table 3). There were significant correlations between the fusiform, ITG and MTG ROIs. Importantly, given our hypotheses concern the perirhinal cortex, there was no significant correlation between any of these ROIs and the PRc ROI. Although there was a positive relationship the PRc and fusiform ROIs, the low Pearson correlation score shows that there is distinct variance in the damage scores for each of these two ROIs.

Performance for the two patient groups was compared to each other, in addition to comparisons with healthy controls. Further, to test whether damage to specific regions within the anterior temporal lobes (such as the PRc) has a distinct effect on semantic processing we also test for the relationship between the structural integrity of anterior temporal ROIs to performance in the different experiments.

### 3.2 Experiment 1: Picture naming

#### 3.2.1 1a – Object category analysis

**Fig. 4:**
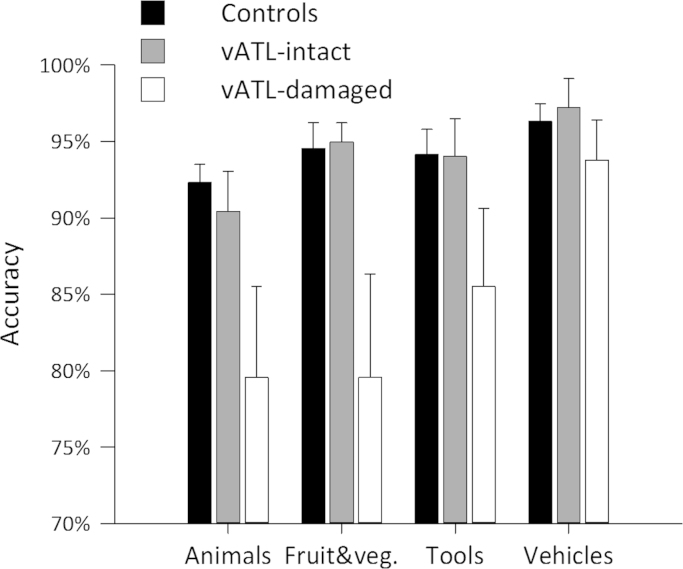
Mean picture naming accuracy for each group and category (error bars: SEM).

We first tested whether our three participant groups (vATL-damaged, vATL-intact and healthy controls) showed any difference in accuracy when naming pictures of objects from different categories. Overall naming accuracy for each group and each participant is presented in Table 4 and Fig. 4, showing that the vATL-damaged group is less accurate and more variable compared to the other groups (see Table S3 for the accuracies of the vATL-damaged group according to hemisphere).

To test for differences in accuracy across the three groups and for different object domains (i.e. living and nonliving things) we performed a 3 (group)×2 (domain) ANOVA . The ANOVA showed a significant main effect of group (*F*(2,26)=4.50, *p*=0.021) driven by worse overall performance for the vATL-damaged group compared to the other two groups. There was no effect of domain and no domain by group interaction (both *F*'s<1.5; *p*'s>0.2). Planned comparisons between accuracy for living and nonliving objects for each group revealed that only the vATL-damaged group showed significantly lower accuracy on living compared to nonliving objects (correct: living 81%, nonliving 85%, *t*(7)=2.88, *p*=0.024).

To investigate the effect of damage on naming accuracy in more detail, we performed an additional 3 (group)×4 (object category) ANOVA . As well as main effects of group (*F*(2,26)=4.63; *p*=0.019) and object category (*F*(3,78)=9.26; *p*<0.001), the ANOVA revealed a significant interaction between group and category (*F*(6,78)=2.52, *p*=0.028) due to differential performance across the three groups for the different categories. Additional one-way ANOVAs for each category showed a significant group effect for animals (*F*(2,26)=4.59, *p*=0.020) and fruit and vegetables (*F*2,26)=5.37; *p*=0.01) but not for the two nonliving categories (Tools: *F*(2,26)=2.5, *p*=0.1; Vehicles: *F*<1). Post-hoc least significant difference tests revealed these effects were driven by significantly worse performance for the vATL-damaged group compared to both controls (animals, difference=13% *p*=0.006; *F* and V, difference=15%, *p*=0.005; compared with tools, difference=9%, *p*=0.043; vehicles, difference=3%, *p*=0.3) and the vATL-intact group (animals, difference=11%, *p*=0.05; *F* and V, difference=15%, *p*=0.016; compared with tools, difference=9%, *p*=0.1; vehicles, difference=3%, *p*=0.26). There were no significant differences between the vATL-intact group and controls (maximum difference=2%, all *p*'s>0.6). Finally, paired *t*-tests between categories for the vATL-damaged group revealed that performance was significantly worse for animals compared to tools (6% difference, *t*(7)=4.14, *p*=0.004) and vehicles (14% difference, *t*(7)=3.92, *p*=0.006) while fruit and vegetables were less accurately identified than vehicles (14% difference, *t*(7)=3.06, *p*=0.018). Although note that there was also a significant 8% difference between tools and vehicles (*t*(7)=2.84; *p*=0.025) and no difference between fruit and vegetables and tools (*t*(7)= 1.84; *p*>0.1). These results show that the vATL-damaged group have significantly reduced naming accuracy for living things (including animals and fruit and vegetables) compared to other object categories, and reduced accuracy for living things compared to healthy controls and other temporal lobe damaged patients.

**Fig. 5:**
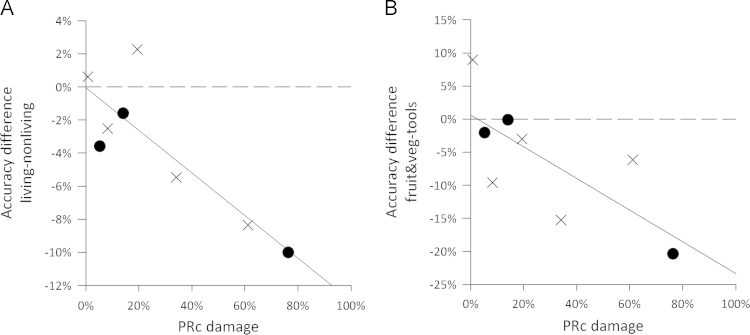
Relationship between regional damage and performance. Greater PRc damage predicts larger accuracy differences for A: living–nonliving objects, and B: Fruit and veg-tools. Crosses denote left hemisphere lesions and circles right hemisphere.

To test how damage to different regions within the anterior temporal lobe influences naming accuracy, we correlated various accuracy measures with the degree of damage in each anterior temporal lobe ROI (Table 5, Fig. 5). Spearman's rank correlations showed that damage to the perirhinal cortex was significantly correlated with the difference between accuracy for living and nonliving objects (Spearman's rho=−0.67, *p*=0.035), showing that increased damage to the PRc is associated with larger differences in performance for living compared to nonliving objects. This effect remained significant after controlling for damage in the neighbouring fusiform ROI using partial Spearman's rank correlation (Spearman's rho=−0.72, df=5, *p*=0.035). A similar relationship was found between damage in the perirhinal cortex and the difference in accuracy between fruit and vegetables and tools (Spearman's rho=−0.76, *p*=0.014). No other ROIs significantly correlated with performance.

In summary, these results show that damage to the anterior temporal lobes results in poorer naming accuracy for living things compared to healthy controls and patients with damage to other temporal lobes structures. Critically, we also show that damage to the perirhinal cortex within the anterior medial temporal lobes correlates with the degree of reduced performance for living compared to nonliving objects.

#### 3.2.2 1b – Conceptual structure analysis

In the previous analysis we tested the relationship between performance for different object categories and damage. Here we test a more specific hypothesis, based on the conceptual structure account, that putative category and domain effects can be explained by the differing conceptual structure properties that are typically associated with objects from different categories. We calculated three key measures from our property norm data (Devereux et al., 2014) that quantify the internal conceptual structure of different objects (see Section 1). Based on the CSA and our previous findings (e.g. Moss et al., 2005; Tyler et al., 2013) we predict that damage to the ventral anterior temporal lobe, in particular the perirhinal cortex, will impair the ability to differentiate between objects with many shared and few, weakly correlated distinctive properties, such as living things. This is captured by the ‘correlation×distinctiveness’ measure in which high values relate to concepts whose distinctive properties are more highly correlated (typically tools) and lower values for concepts whose more shared properties are more highly correlated (typically animals). We also tested for the influence of a concept's correlational strength and mean distinctiveness on naming accuracies.

**Fig. 6:**
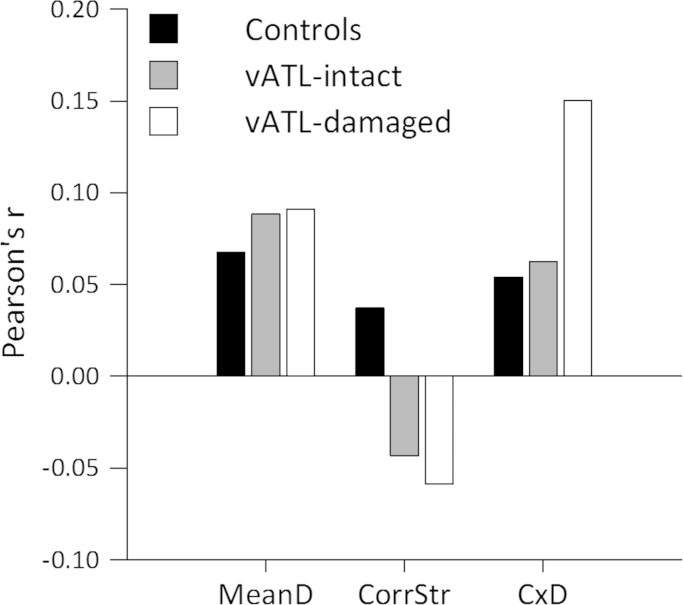
Conceptual structure analysis. Item-wise Pearson's correlations between group accuracy and the conceptual structure statistics.

We first calculated the mean percent correct naming response for each object and for each of the three groups, before testing the relationship between accuracy and the three conceptual structure measures. We performed separate ANCOVAs for each conceptual structure measure. We found a marginally significant interaction between group and the ‘correlation×distinctiveness’ measure (*F*(2,410)=2.98, *p*=0.052) showing differential correlations between accuracy and ‘correlation×distinctiveness’ across the three groups. Neither of the other two variables showed a comparable interaction effect (Mean distinctiveness; *F*(2,410)<2, Correlational strength; *F*(2,410)<2). Post hoc correlations for each group (Table 6, Fig. 6, see Table S4 for the vATL-damaged group according to hemisphere) showed that the ‘correlation×distinctiveness’ measure correlated with accuracy for the vATL-damaged group only (*r*=0.15, *p*=0.015), and furthermore correlated with the difference in accuracy between the vATL-damaged group and controls (*r*=0.14, *p*=0.023) and the difference between the vATL-damaged and vATL-intact groups (*r*=0.14, *p*=0.026). The vATL-damaged group performed relatively better for objects whose distinctive properties are more highly correlated (for example, tools) compared to objects whose more shared properties are more highly correlated (for example, animals). In contrast, the other two groups' performance was unrelated to this measure of semantic complexity. These effects are consistent with the categorical effects reported in Section 3.2.1. As objects with highly correlated shared properties tend to be more confusable, this suggests that the vATL-damaged group had most difficultly accurately naming objects that are more confusable with other members of the same category.

**Fig. 7:**
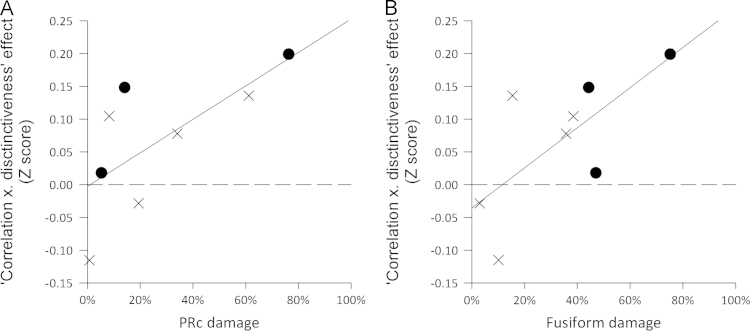
Relationship between the damage and the correlation of accuracy and ‘correlation×distinctiveness’ in A: the perirhinal cortex, and B: in the fusiform. Crosses denote left hemisphere lesions and circles right hemisphere.

To determine which specific regions within the ATL underpin the ‘correlation×distinctiveness’ effect in the vATL-damaged group, we correlated damage in the ROIs with the correlation between accuracy and the three conceptual structure variables (Table 7, Fig. 7). Each participant's variable×accuracy effect was calculated using Pearson correlation followed by Fisher transformation to give a Z score. We found that degree of damage to the perirhinal cortex, and to the adjacent anterior fusiform, were significantly correlated with each participant's correlation between accuracy and ‘correlation×distinctiveness’ (PRc, Spearman's rho=0.67, *p*=0.035; anterior fusiform, Spearman's rho=0.67, *p*=0.035). Partial Spearman's rank correlations showed that the relationship with perirhinal cortex remained significant after controlling for damage in the anterior fusiform, and vice versa. These effects show that more damage in these regions is associated with a more positive correlation between accuracy and ‘correlation×distinctiveness’. This relates to poorer accuracy for objects with lower values of ‘correlation×distinctiveness’ (typically animals) compared to accuracy on objects with high values on ‘correlation×distinctiveness’ (typically tools). The fusiform ROI also correlated with the effect of mean distinctiveness. The mean distinctiveness variable encodes category-level information, which suggests that damage to the anterior fusiform affects more general semantic processing as well as processing of semantically complex objects. In contrast there is no correlation between PRc and mean distinctiveness, indicating a more specialised role for this region.

In summary, the results from the conceptual structure analysis extend our findings from the category analysis to show that the vATL-damaged group show reduced naming accuracy for objects that have specific conceptual structure properties – namely worse performance for objects whose more shared properties are more highly correlated than objects with more distinctive correlated properties. Crucially, we showed that the relationship between ‘correlation×distinctiveness’ and accuracy was most strongly influenced by damage to the perirhinal cortex and adjacent anterior fusiform where the greater the degree of damage to these regions the worse participants performed for items with low ‘correlation×distinctiveness’ values. Only the PRc correlated selectively with the ‘correlation×distinctiveness’ effect, whereas damage to the anterior fusiform also influences the effect of mean distinctiveness.

### 3.3 Experiment 2: Word–picture matching

**Fig. 8:**
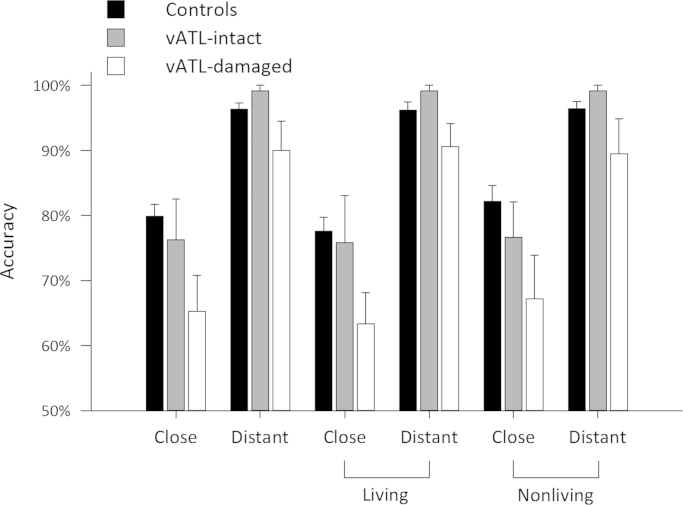
Word-picture matching analysis. Group mean accuracy for the close and distant conditions in the word-picture matching task, for all items combined and for living and nonliving items separately.

While experiment 1 aimed to uncover the categorical and conceptual structure underpinnings of semantic impairments in patients with damage including the perirhinal cortex, here we test the extent to which these patients also have increased difficulty when making distinctions between semantically similar items. Participants carried out a word-picture matching task in which we manipulated the relationship between the word and picture such that they were either semantically similar (close condition) or semantically distant (distant condition) where close and distant were defined by semantic feature overlap in our property norms (see Section 2). Words/pictures were either living or nonliving items. We predicted that patients with damage in the anterior temporal lobe would have more difficulty with the similar word/object pairings than the other two groups, and that such effects will be most strongly associated with damage to the perirhinal cortex. Accuracy for each group of participants, and the individual scores are shown in Table 8 and Fig. 8 (see Table S5 for the accuracies of the vATL-damaged group according to hemisphere).

To test for differences in accuracy between close and distant judgements across the three groups and for different object domains, we performed a 3 (group)×2 (domain)×2 (distance) ANOVA. There were significant main effects of group (*F*(2,21)=4.74, *p*=0.02) and distance (*F*(1,21)=140, *p*<0.001), with a marginal group by distance interaction (*F*(2,21)=2.85, *p*=0.08) suggesting differences between groups in the semantic distance effect. There was no effect of domain (*F*(1,21)=1.19, *p*=0.29), no interaction between domain and group nor between domain, group and distance (both *F*'s<1). The interaction between domain and distance was not significant (*F*(1,21)=2.14, *p*=0.16).

Follow-up paired *t*-tests showed that all groups were significantly worse for close pairs compared to distant pairs (controls, difference=16%, *t*(13)=14.04, *p*<0.001; vATL intact, difference=23%, *t*(3)=3.67, *p*=0.035; vATL damaged, difference=25% *t*(5)=0.002), with the vATL-damaged group showing the largest difference in accuracy between close and distant pairs. Further, two-sample *t*-tests comparing accuracy between groups showed that the vATL-damaged group were significantly less accurate than controls on close pairs (difference=15%, *t*(18)=3.25, *p*=0.002). There was no significant difference between vATL-damaged and vATL-intact patients (*t*(8)=1.29, *p*=0.12), although there was an appreciable numeric difference in mean accuracy of 11% (*t*(8)=1.3, *p*=0.12). No differences were seen between the vATL-intact group and healthy controls (*t*<1). For the distant items, the small group of vATL-intact patients made fewer errors than the controls (difference=3%), resulting in no significant difference between the vATL-damaged group and controls (difference=6%, *t*(5.5)=1.38, *p*>0.1), but a difference between the vATL-damaged and vATL-intact groups (difference=9%, *t*(5.3)=2.0, *p*=0.05). Finally, the distance effect (close–distant pairs) was marginally greater for the vATL-damaged group compared to controls (*t*(5.78)=1.88, *p*=0.055), with no differences observed between other groups (both *t*'s ≤1). Taken together, these results show that the group who have vATL damage showed the poorest performance when needing to distinguish between semantically similar items (i.e. poorest performance on close items, and biggest difference between close and distant items). Successful performance on the close pairs will place demands on the conceptual processing of distinctive information, as the semantically similar close pairs have a large degree of shared feature information in common. This may imply that the vATL-damaged group show impaired processing of distinctive feature information that is required to distinguish between otherwise similar objects.

**Fig. 9:**
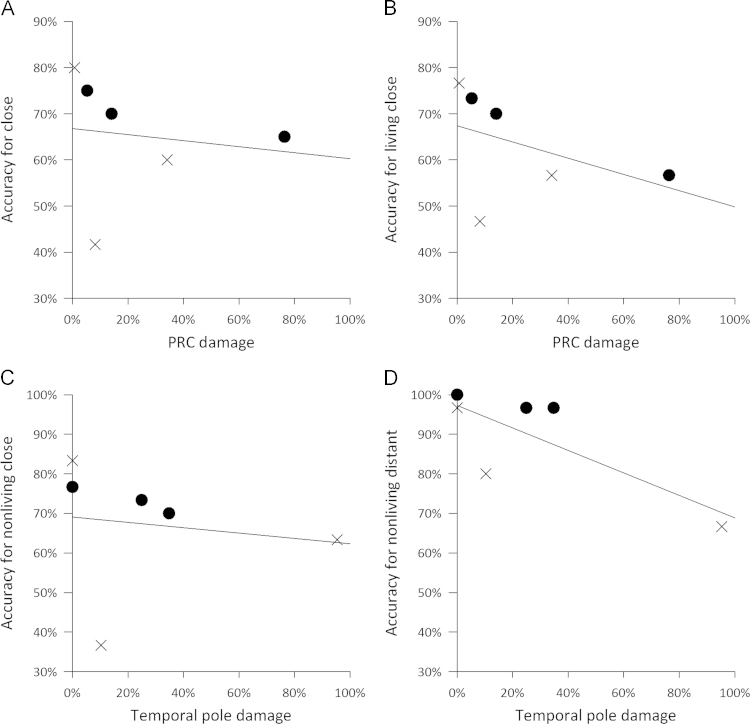
Relationships between ROI damage and word-picture matching accuracy. PRc damage correlated with accuracy for A: close items, and B: living close items at a trend level. Trend-level correlations with temporal pole damage and C: nonliving close items and D: nonliving distant items.

Finally, to determine if damage to specific regions within the anterior temporal lobes is differentially contributing to impaired performance in the vATL-damaged group, we correlated damage in the ROIs with accuracy (Table 9, Fig. 9). There was a trend for a negative correlation between damage in the PRc and accuracy for the close items (Spearman's rho=−0.60, *p*=0.10) that was marginally significant for the living close items (Spearman's rho=−0.64, *p*=0.087), showing that increasing damage to the perirhinal cortex results in poorer performance on close items, especially of living things (Fig. 9A, B). These relationships remained at trend level after controlling for damage in the neighbouring fusiform ROI. Last, there was a marginal relationship between damage in the temporal pole and accuracy for both nonliving close (Spearman's rho=−0.64, *p*=0.087) and distant pairs (Spearman's rho=−0.65, *p*=0.083). Aside from this, there were no significant correlations with accuracy for distant pairs.

Overall, the results from Experiment 2 show that damage to the perirhinal cortex results in poorer performance when differentiation between semantically similar items is required. The vATL-damaged group showed the largest semantic distance effect and worst accuracy on the close items. Critically, accuracy for close items was also correlated with the extent of damage to the perirhinal cortex, while the temporal pole showed a relationship to performance on nonliving items regardless of semantic distance. Together, these results contribute to the evidence that damage to the perirhinal cortex results in the impaired processing of distinctive feature information that is required to distinguish between otherwise similar objects.

## 4 Discussion

In two experiments, we investigated the role of the PRc in fine-grained semantic processing. We tested a group of patients with damage to the PRc, and other vATL subregions, and compared their performance with two other groups – one lesion-free and the other with damage to ventral stream regions, but sparing the PRc. Our main findings were that greater damage to the PRc resulted in worse performance at (1) naming pictures of living things, (2) naming objects requiring the most fine-grained semantic integration (i.e., those with low values on the ‘correlation×distinctiveness’ measure), and (3) correctly rejecting semantically confusable words and pictures. Furthermore, these effects were not consistently associated with any other ATL subregion across the experiments. Together, these results show that the degree of damage to the PRc is related to worse performance for items that require fine-grained semantic processing.

The present study provides converging support for the role of the PRc in conceptual processing when complex semantic information needs to be integrated. Throughout our results we consistently found a relationship between the PRc and performance for more semantically confusable items across the experiments, and was not consistently observed in other ventral and more lateral subregions of the ATL. The PRc is considered to sit at the apex of the ventral visual pathway (Bussey and Saksida, 2002; Murray and Bussey, 1999; Murray and Richmond, 2001) and also receives uni- and poly-modal inputs from other sensory regions (Libby et al., 2012; Suzuki and Amaral, 1994). A large body of evidence suggests a critical role for the PRc in processing complex conjunctions of information enabling fine-grained distinctions between perceptually ambiguous items, where behavioural responses cannot be guided by single object features, but require conjunctive processing of multiple features (Barense et al., 2007, 2012; Buckley et al., 2001; Bussey and Saksida, 2002; Murray and Bussey, 1999; Murray and Richmond, 2001). In addition to processing perceptual complexity, the PRc has also been implicated in similar functions for conceptual processes, where more fine-grained conceptual processing is needed when concepts are more confusable (Barense et al., 2010; Clarke and Tyler, 2014; Kivisaari et al., 2012; Moss et al., 2005; Taylor et al., 2006, 2009; Tyler et al., 2013).

The results of the current study are particularly important as converging evidence for the role of the PRc in conceptual processing for more semantically confusable categories (animals and fruits/vegetables) and for concepts whose conceptual structure results in the more distinctive properties being more difficult to integrate into the representation (low values of ‘correlation×distinctiveness’). By showing a consistent relationship between the severity of damage to the PRc and performance for semantically confusable concepts, we show the necessity of this region in this cognitive function. Further, our results showed a relationship between performance on the semantically close items (high semantic confusability) for the word-picture matching task and damage including the PRc. Although damage to the anterior fusiform gyrus also related to impaired processing of concepts with low ‘correlation×distinctiveness’, damage also impaired processing of concepts whose features were less distinctive, whereas damage to PRc did not. The mean distinctiveness variable reflects category-level information that is represented earlier in the processing hierarchy. This finding supports the hypothesis that only the PRc is specialised in processing more complex semantic feature conjunctions and also raises the possibility that the ‘correlation×distinctiveness’ impairment following fusiform damage is reflects downstream processing difficulties. However, the anterior fusiform is also strongly linked to conceptual processing in general (e.g. Binney et al., 2010 2012; Lambon Ralph et al., 2010; Mion et al., 2010; Visser et al., 2010) and here we provide further evidence for this, in addition to highlighting the important dissociable cognitive functions of the PRc.

The effects relating PRc damage to the semantically close items only showed statistical trends, with a stronger effect when only considering living close items. Therefore the cosine similarity measure that was used to define the ‘close’ and ‘distant’ pairs, and was used to match living and nonliving pairs, cannot entirely account for semantic confusability. Closer examination of the stimuli in the word-picture matching experiment shows that there is a difference between the number of shared features for living (mean=12.7) and nonliving items (mean=11.1, *p*<0.05), even though overall the number of features was matched. This suggests that for living things there is greater activation of multiple correlated features, thereby increasing the confusability of the living item pairs compared to the nonliving pairs. Measures for the distinctiveness by correlation interaction also show that the living things in the word picture matching experiment have lower values than nonliving things, similar to the naming experiment. Although effects relating PRc damage to the semantically close items only showed statistical trends, the effects observed are consistent with the hypothesis that the PRc acts to process more semantically confusable concepts, and resonates with reports showing patients with damage in the MTL, including the PRc, are impaired in visual discrimination tasks for which there is high perceptual feature overlap (Barense et al., 2005, 2007, 2012).

Along with the PRc, damage to the temporal poles also showed a relationship to performance in the word picture matching task. This task, in addition to distinguishing between the semantics of two concepts, also involves integrating information across modalities. Both the temporal pole and PRc can integrate information from multiple modalities (Binder and Desai, 2011; Duffau et al., 2013; Taylor et al., 2009). However, the temporal pole would appear to be interested in integration of information more generally, as there was no influence of semantic distance on the correlations, while the PRc showed differential effects across the conditions with only the close conditions showing an effect, indicating that semantic confusability is driving the effects in this region. Overall, our results provide further evidence that the PRc is involved not only in purely perceptual processing, but also in the domain of conceptual processing. This is consistent with the hypothesised general representational role of the PRc in representing complex conjunctions of information that is relevant to the behavioural response, as in the representational hierarchy theory (Cowell et al., 2010).

The majority of research on semantic memory and its relationship to the ATL has been conducted with SD patients (e.g. Hodges et al., 1992; Lambon Ralph et al., 2010; Patterson, 2007; Rogers et al., 2004; Rogers and Patterson, 2007) , whose damage is bilateral, extensive (including all of the ATL and often other regions as well) and progressive. Research based on this disorder has clearly provided evidence for ATL involvement in semantic memory. However, because of the widespread nature of the damage, it is difficult to determine whether specific substructures within the ATL that underpin the patients' semantic deficits. The patients in the current study were stable with single unilateral lesions with varying amounts of damage across ATL subregions. This does not completely overcome the fundamental limitation of lesion studies that naturally occurring lesions do not respect anatomical boundaries. However, the variation in damage across regions has allowed us to test whether the range of performance in different tasks is associated with the extent of damage to different subregions within the ATL. The consistent finding was that PRc damage, regardless of affected hemisphere, was associated with poorer performance for items that are more semantically confusable and that require more fine-grained semantic processing. This remained true even when amount of damage to neighbouring regions, such as the fusiform, were accounted for. By combining a detailed anatomical approach where we correlate performance with varying degrees of damage across regions, we have been able to provide key evidence for the necessary role of the PRc in conceptual processing that converges with evidence from functional neuroimaging studies with non-impaired participants (Clarke and Tyler, 2014; Moss et al., 2005; Taylor et al., 2006; Tyler et al., 2013; Wang et al., 2010) and patient studies where detailed anatomical approaches have been adopted (Davies et al., 2004; Kivisaari et al., 2012).

None of the patients with perirhinal damage reported here showed a global deficit in semantic memory. Patients showed comparable performance to controls for tools and vehicles, and for effects of mean distinctiveness and correlational strength in the naming study, and for the semantically distant items in the word picture matching task. In contrast, their deficits were most prominent for the most semantically challenging items showing a disproportionate dependence on more medial structures within the ATL.

Two factors may help to explain the dependence on the PRc seen here. First, the more lateral and medial aspects of the ATL could play different roles in semantic cognition due to the computational properties of these regions, with the more lateral ATL regions (e.g. IT) supporting general semantic processes and the more medial aspects (the PRc) supporting fine-grained semantic processing. This notion has been previously suggested based on comparisons of patient populations (Moss et al., 2005; Noppeney et al., 2007), and may be underpinned by the computational capacities of the regions with the PRc strongly implicated in processes that require fine-grained perceptual and semantic distinctions to be made. Further, the perirhinal cortex is believed to be involved in feedback signals to more posterior regions (Miyashita et al., 1996). Supporting this, Campo et al. (2013) reported reduced feedback connectivity from the ATL to the posterior ventral temporal cortex for patients with highly focal lesions, some of which were in the PRc. Increasing damage to the PRc in our patients was associated with worse performance which may suggest a breakdown in the feedback mechanisms that may be required for successful feature binding. In contrast, the lateral ATL is one component of the default mode network (DMN) that has been suggested to support semantic processing (Binder et al., 2009, 1999), and may do so when semantic demands are relatively low. A second factor is that both experiments reported here involved visual images that may lead to increased dependence on more medial aspects of the ATL in contrast to the more lateral focus that may be seen with language input (Visser et al., 2010). Such medial-lateral distinctions could be underpinned by the differential connectivity of lateral and medial regions with the ventral language and visual pathways respectively (see Binney et al., 2012), however there is also evidence that the PRc and other MTL structures support cross-modal integration of complex semantic information (Quian Quiroga et al., 2009; Taylor et al., 2006, 2009). As such, these regional semantic distinctions in the ATL do not suggest a unitary amodal hub, but instead suggest there are computational and/or modality-dependent biases across the ATL underpinned by divergent anatomical connectivity.

Given the prominent role attributed to the ATL in semantic memory, a more detailed understanding of the differential neurocognitive functioning across the area is needed. By studying a group of patients with variable damage across the ATL, and quantifying the degree of damage across different anatomically defined subregions, we have been able to show that the PRc, in the medial aspect of the ATL, provides a necessary and crucial neurocognitve function. By relating behavioural performance to the structural integrity of a range of ATL subregions, we have been able to show the importance of the perirhinal cortex in supporting fine-grained semantic processes across different tasks – picture naming and word-picture matching. Our results support the notion that the PRc is the primary structure within the ATL that is necessary to support fine-grained conceptual processes. Further, the relationship between damage and our specific measures of performance support a distributed feature-based semantic system where the PRc acts to process the most complex conjunctive representations to support conceptual processes.

## Figures and Tables

**Table 1:** Patients' demographic and lesion information.

**ID**	**Group**	**Sex**	**Age at onset**	**Years to first test**	**Aetiology**	**Lesion**	**Lesion volume (cm**^**3**^**)**
P1	vATL-damaged	F	54.8	7.5	Excision of meningioma	L MTG and temporal pole, extending to PRc	12.9
P2	vATL-damaged	M	59.6	11.3	Haematoma	L anterior temporal white matter, extending to ITG, fusiform and PRc	18.4
P3	vATL-damaged	M	41.0	7.0	Abscess	L middle and inferior temporal gyri	5.8
P4	vATL-damaged	M	52.7	9.7	Excision of meningioma	L temporal, ITG, fusiform and PRc anterior to hippocampus and part of MTG	27.0
P5	vATL-damaged	M	36.0	11.8	AV malformation and haematoma	R fusiform, ITG & MTG, extending to temporal pole and white matter overlying PRc	15.5
P6	vATL-damaged	M	43.0	13.0	Resection for epilepsy	Entire R temporal lobe anterior to body of hippocampus, sparing part of superior temporal gyrus only.	17.5
P7	vATL-damaged	F	46.7	21.4	Meningioma	LPRc & fusiform	12.6
P8	vATL-damaged	F	27.6	3.1	Excision of tumour	R temporal pole , fusiform, PRc and part of ITG	10.0
P9	vATL-intact	M	40.0	4.8	Haemorrhagic stroke (post-surgical)	R dorsal temporal pole and superior temporal gyrus.	4.6
P10	vATL-intact	F	19.7	15.2	Ischaemic stroke	R posterior ITG.	6.8
P11	vATL-intact	M	32.8	4.8	Glioma	L medial occipital cortex.	12.8
P12	vATL-intact	M	42.5	17.5	Ischaemic stroke	R medial occipitotemporal cortex.	42.0
P13	vATL-intact	F	47.6	4.5	Ischaemic stroke	R medial occipital cortex.	9.7
P14	vATL-intact	M	22.6	5.5	Cavernoma	L posterior ITG.	8.5

**Table 2:** ROI borders.

**Region**	**Anterior**	**Medial**	**Lateral**	**Posterior**
Temporal pole	Anterior tip of temporal lobe	Fundus of temporopolar sulcus	Superior or inferior temporal sulcus	3 mm anterior to grey matter of the limen insulae
Perirhinal cortex	2 mm anterior to grey matter of the limen insulae	Shoulder of medial bank of collateral sulcus	Shoulder of lateral bank of collateral sulcus^a^	3 mm posterior to apex of intralimbic gyrus
Entorhinal cortex	2 mm posterior to white matter of the limen insulae	Most medial extent of parahippocampal gyrus	Shoulder of medial bank of collateral sulcus	1 mm posterior to apex of intralimbic gyrus
Fusiform gyrus	Anterior limit of occipitotemporal sulcus	Shoulder of lateral bank of collateral sulcus^a^^,^^b^	Occipitotemporal sulcus	3 mm posterior to apex of intralimbic gyrus
Inferior temporal gyrus	Anterior limit of inferior temporal sulcus	Inferior temporal sulcus	Superior temporal sulcus	3 mm posterior to apex of intralimbic gyrus
Middle temporal gyrus	Anterior limit of inferior temporal sulcus	Occipitotemporal sulcus	Inferior temporal sulcus	3 mm posterior to apex of intralimbic gyrus

a This border varied according to the depth of the collateral sulcus (Insausti et al., 1998; Kivisaari et al., 2013).

b This definition is taken from Insausti et al. (1998) and differs from Tzourio-Mazoyer et al. (2002).

**Table 3:** Correlations between % ROI damage for pairs of anterior temporal ROIs.

	**MTG**	**ITG**	**Fusiform**	**PRc**
**Tpole**	−0.05	−0.10	0.01	0.16
**PRc**	0.04	−0.08	0.39	
**Fusiform**	**0.54**	**0.85**		
**ITG**	**0.72**			

Bold: *p*<0.05.

**Table 4:** Picture naming accuracy for different object categories.

**Initials**	**Patients**	**All (%)**	**Living (%)**	**Non-living (%)**	**Animal (%)**	**Tool (%)**	**Fruit and veg (%)**	**Vehicle (%)**
P1	vATL-damaged	98	99	97	100	100	97	100
P2	vATL-damaged	46	45	47	42	52	42	78
P3	vATL-damaged	92	92	92	85	88	97	100
P4	vATL-damaged	87	84	90	85	88	73	94
P5	vATL-damaged	94	92	96	91	96	94	100
P6	vATL-damaged	79	73	83	79	84	64	94
P7	vATL-damaged	85	80	88	79	88	82	89
P8	vATL-damaged	84	83	85	76	88	88	94
P9	vATL-intact	98	98	98	97	100	97	100
P10	vATL-intact	95	96	95	94	96	94	89
P11	vATL-intact	88	90	87	88	84	94	100
P12	vATL-intact	92	89	95	79	92	94	100
P13	vATL-intact	95	97	93	94	92	100	100
P14	vATL-intact	94	92	95	91	100	91	94
Mean	vATL-damaged	83	81	85	80	86	80	94
	vATL-intact	94	93	94	90	94	95	97
	Controls	94	94	94	92	94	95	96
								
**SEM**	vATL-damaged	5.7	5.9	5.6	6.0	5.1	6.8	2.7
	vATL-intact	1.3	1.5	1.5	2.6	2.5	1.3	1.9
	Controls	0.7	1.3	0.9	1.2	1.7	1.7	1.2

**Table 5:** Spearman's rank correlations between ROI damage and picture naming scores.

	**T pole**	**PRC**	**Fusiform**	**ITG**	**MTG**
Living–nonliving	0.04	**−0.67**	−0.60	−0.33	−0.10
Animal–tool	0.15	−0.08	−0.55	−0.35	0.06
Animal–vehicle	0.11	0.05	−0.40	−0.36	0.07
Fruit and veg–vehicle	−0.22	−0.48	−0.54	−0.37	−0.14
Fruit and veg–tool	−0.46	**−0.76**	−0.33	−0.21	−0.31

Bold: *p*<0.05.

**Table 6:** Pearson's correlations between naming accuracy and conceptual structure measures.

**Variable**	**Controls**	**vATL-intact**	**vATL-damaged**	**vATL-intact vs. Controls**	**vATL-damaged vs. Controls**	**vATL-damaged vs. vATL-intact**
Mean distinctiveness	0.07	0.09	0.09	0.03	0.06	0.04
Correlational strength	0.04	−0.04	−0.06	−0.09	−0.10	−0.04
‘Correlation×distinctiveness’	0.05	0.06	**0.15**	0.01	**0.14**	**0.14**

Bold: *p*<0.05

**Table 7:** Spearman's rank correlations between ROI damage and relationship between accuracy and conceptual structure variables.

**Variable**	**T pole**	**PRC**	**Fusiform**	**ITG**	**MTG**
Mean distinctiveness	−0.05	0.38	**0.67**	0.48	0.02
Correlational strength	−0.60	−0.02	0.21	0.02	0.00
‘Correlation×distinctiveness’	0.24	**0.67**	**0.67**	0.45	0.19

Bold: *p*<0.05.

**Table 8:** Accuracy in the word-picture matching task for the close and distant conditions.

**Initials**	**Group**	All items		Living		Nonliving	
		**Close (%)**	**Distant (%)**	**Close (%)**	**Distant (%)**	**Close (%)**	**Distant (%)**
P2	vATL-damaged	42	82	47	83	37	80
P3	vATL-damaged	80	95	77	93	83	97
P4	vATL-damaged	60	72	57	77	63	67
P5	vATL-damaged	75	98	73	97	77	100
P6	vATL-damaged	65	95	57	93	73	97
P8	vATL-damaged	70	98	70	100	70	97
P9	vATL-intact	90	100	90	100	90	100
P10	vATL-intact	75	97	73	97	77	97
P12	vATL-intact	60	100	57	100	63	100
P14	vATL-intact	80	100	83	100	77	100
**Mean**	vATL-damaged	65	90	63	91	67	89
	vATL-intact	76	99	76	99	77	99
	Controls	80	96	78	96	82	96
							
**SEM**	vATL-damaged	6	4	5	4	7	5
	vATL-intact	6	1	7	1	5	1
	Controls	2	1	2	1	2	1

**Table 9:** Spearman's rank correlations between ROI damage and word-picture matching accuracy.

	**T pole**	**PRC**	**Fusiform**	**ITG**	**MTG**
Close	−0.58	−0.60	−0.09	−0.09	−0.26
Distant	−0.39	−0.29	0.50	0.50	0.29
Living close	−0.53	−0.64	−0.20	−0.20	−0.38
Living distant	−0.28	−0.23	0.46	0.46	0.23
Nonliving close	−0.64	−0.49	0.03	0.03	−0.09
Nonliving distant	−0.65	−0.39	0.52	0.52	0.39
