# Supplementary material for: The perirhinal cortex and conceptual processing: Effects of feature-based statistics following damage to the anterior temporal lobes
Source: Neuropsychologia. 2015 Sep;76:192–207. doi: 10.1016/j.neuropsychologia.2015.01.041 (PMC4582809; doi:10.1016/j.neuropsychologia.2015.01.041)
Supplement: Supplementary file 1 — Supplementary Material [file mmc1.doc]

**Supplementary tables**

Table S1. Stimulus information for 4 different object categories within the set of 207 objects.

|  |  |  | **Agreement** | |
| --- | --- | --- | --- | --- |
|  | **Familiarity** | **Exemplarity** | **Name** | **Concept** |
| Animals | 3.76 | 5.36 | 92.23 | 93.86 |
| Fruit & veg | 5.02 | 5.82 | 93.40 | 93.86 |
| Tools | 4.67 | 5.31 | 92.92 | 95.58 |
| Vehicles | 4.77 | 5.41 | 86.10 | 93.87 |

Table S2: Descriptive statistics of the different items in word-picture matching. Matching by condition (close and distant) and domain (living and nonliving)

|  |  |  |  | Word descriptives | | Picture descriptives | |
| --- | --- | --- | --- | --- | --- | --- | --- |
|  |  |  | cosine similarity | familiarity | letters | exemplarity | visual familiarity |
| Living | close | Mean | 0.69 | 474 | 6.3 | 5.3 | 4.1 |
|  |  | sd | 0.10 | 59 | 2.0 | 0.7 | 1.4 |
|  |  | Median | 0.67 | 487 | 6.0 | 5.3 | 4.2 |
|  | distant | Mean | 0.20 | 484 | 6.3 | 5.2 | 3.7 |
|  |  | sd | 0.09 | 56 | 2.5 | 0.8 | 1.3 |
|  |  | Median | 0.20 | 503 | 5.0 | 5.2 | 3.5 |
| Nonliving | close | Mean | 0.70 | 497 | 5.7 | 5.3 | 4.2 |
|  |  | sd | 0.08 | 65 | 1.9 | 0.8 | 1.4 |
|  |  | Median | 0.69 | 497 | 5.0 | 5.4 | 4.2 |
|  | distant | Mean | 0.20 | 502 | 5.8 | 4.9 | 3.8 |
|  |  | sd | 0.09 | 91 | 2.2 | 1.0 | 1.5 |
|  |  | Median | 0.21 | 517 | 5.0 | 5.2 | 4.1 |

Table S3. Picture naming accuracy for different object categories for the vATL-damaged patients according to hemisphere

| **Hemisphere** | **all** | **living** | **non-living** | **animal** | **tool** | **fruit & veg** | **vehicle** |
| --- | --- | --- | --- | --- | --- | --- | --- |
| Left | 82% | 80% | 83% | 78% | 83% | 78% | 92% |
| Right | 86% | 83% | 88% | 82% | 89% | 82% | 96% |

Left: n=5. Right: n=3.

**Table S4. Pearson’s correlations between naming accuracy and conceptual structure measures for the vATL-damaged patients according to hemisphere**

| **Variable** | **Left** | **Right** |
| --- | --- | --- |
| Mean distinctiveness | 0.05 | 0.13 |
| Correlational strength | -0.01 | -0.12 |
| ‘Correlation x. distinctiveness’ | 0.09 | 0.20 |

Left: n=5. Right: n=3.

**Table S5. Accuracy in the word-picture matching task for the close and distant conditions for the vATL-damaged patients according to hemisphere**

|  | All items | | Living | | Nonliving | |
| --- | --- | --- | --- | --- | --- | --- |
| **Hemisphere** | **close** | **distant** | **close** | **distant** | **close** | **distant** |
| Left | 61% | 83% | 60% | 84% | 61% | 81% |
| Right | 70% | 97% | 67% | 97% | 73% | 98% |

Left: n=3. Right: n=3.
